# Tetrodotoxin-Producing Bacteria: Detection, Distribution and Migration of the Toxin in Aquatic Systems

**DOI:** 10.3390/toxins9050166

**Published:** 2017-05-17

**Authors:** Timur Yu. Magarlamov, Daria I. Melnikova, Alexey V. Chernyshev

**Affiliations:** 1 National Scientific Center of Marine Biology, Far Eastern Branch, Russian Academy of Sciences, Vladivostok 690041, Russia; magarlamov.tiu@dvfu.ru (T.Y.M.); melnikova_di@dvfu.ru (D.I.M.); chernyshev.av@dvfu.ru (A.V.C.); 2 School of Biomedicine, Far Eastern Federal University, Vladivostok 690090, Russia; 3 School of Natural Sciences, Far Eastern Federal University, Vladivostok 690090, Russia

**Keywords:** tetrodotoxin, TTX, TTX-producing bacteria, detection, distribution, origin

## Abstract

This review is devoted to the marine bacterial producers of tetrodotoxin (TTX), a potent non-protein neuroparalytic toxin. In addition to the issues of the ecology and distribution of TTX-producing bacteria, this review examines issues relating to toxin migration from bacteria to TTX-bearing animals. It is shown that the mechanism of TTX extraction from toxin-producing bacteria to the environment occur through cell death, passive/active toxin excretion, or spore germination of spore-forming bacteria. Data on TTX microdistribution in toxic organs of TTX-bearing animals indicate toxin migration from the digestive system to target organs through the transport system of the organism. The role of symbiotic microflora in animal toxicity is also discussed: despite low toxin production by bacterial strains in laboratory conditions, even minimal amounts of TTX produced by intestinal microflora of an animal can contribute to its toxicity. Special attention is paid to methods of TTX detection applicable to bacteria. Due to the complexity of toxin detection in TTX-producing bacteria, it is necessary to use several methods based on different methodological approaches. Issues crucial for further progress in detecting natural sources of TTX investigation are also considered.

## 1. Introduction

Tetrodotoxin (TTX) is a non-proteinaceous neurotoxin that blocks voltage-gated sodium (Na+) channels in nerve and muscle tissues. Initial interest in TTX resulted from numerous food poisonings caused by the consumption of TTX-bearing seafood.

In the 1960s, TTX acquired wide popularity as an instrument of experimental neurobiology, although recently wider interest has emerged in using this toxin in therapy for treatment of addiction, epilepsy, as a local and general anesthetic and analgesic, and for other medical purposes [1]. TTX was first discovered in 1909 by Yoshizumi Tahara, who extracted the toxin from the ovaries of globefish (fam. Tetraodontidae), although puffer fish toxicity has been known for a long time [2]. TTX was first isolated in crystalline form in the 1950s [3] and chromatographically extracted in the 1960s [4].

Further investigations found this neurotoxin in a variety of marine and some terrestrial animals and in marine algae [5,6,7,8]. The widespread occurrence of TTX in phylogenetically distinct groups of eukaryotic organisms allows hypothesizing the bacterial origin of the toxin. According to this hypothesis, the symbiotic/associative TTX-producing microflora of TTX-bearing organisms is the initial source of the toxin in the host. However, there are some arguments against the contribution of associative microflora to host organism intoxication. For example, TTX-producing bacteria were found in most but not all TTX-bearing species (see below). Moreover, the level of TTX produced by bacterial strains is much lower than the concentration of this toxin in the host. Detection of TTX-producing bacteria in marine and fresh water sediments allowed hypothesizing TTX bioaccumulation through small zooplankton and detritus feeders along the food chain [9]. In spite of numerous data on TTX-producing bacteria, the contribution of microorganisms to TTX bioaccumulation in marine ecosystems is still subject to debate.

A recent review devoted to TTX-producing marine bacteria [10] emphasized the taxonomic diversity and geographical distribution of TTX-producing microflora associated with marine organisms. However, many issues, such as toxin migration pathways from bacteria into the host animals’ bodies and subsequent toxin bioaccumulation, and the contribution of associated microflora to organism toxicity, were not discussed. A variety of TTX detection methods based on various principles of action results from the complexity of toxin detection in biological samples. The ineffectiveness of some of the detection methods for bacterial samples should be considered before making a conclusion regarding TTX production of in a given strain.

This review focuses on TTX excretion mechanisms of microbial producers and toxin migration from bacteria to host organisms. The ecology and geographical distribution of marine TTX-producing bacteria is also discussed. Special attention is paid to the methods of TTX detection and the reliability of the corresponding data, with special emphasis on methods prone to false positive results for toxin detection.

## 2. Methods for TTX Detection

Currently there are several methodologies for TTX detection in biological samples including bacteria (Table 1). TTX detection methods are divided into three different methodological approaches based on the physicochemical properties of the toxin, its antigen specificity, and neurotoxic effect.

The first widely used methods for TTX detection were bioassays, namely mouse bioassay (MBA) and tissue culture bioassay (TCBA). MBA has been frequently used but due to ethical concerns is banned in most developed countries [11]. Furthermore, a lack of specificity and high individual variability across experimental animals results in low accuracy for this method. TCBA was developed as an alternative to MBA [12]. Bioassay allows evaluation of the toxicity of the sample, but not listing of the individual toxins included.

Highly selective and sensitive immunological methods (for example ELISA [13]) employing antibodies against TTX are rarely used; however these allow determination of both the presence of the toxin and its visualization inside the cells. It has been shown that antibodies against TTX might have cross-reactivity to saxitoxin (STX) and TTX analogues [14,15]. In 1991, Kaufman et al. produced rabbit anti-TTX antiserum that cross-reacted with both TTX and STX [14]. The cross-reactivity of antibodies against TTX to STX may be associated with the same epitope, which is recognized by antibodies. Both toxins shows remarkable stereospecific similarities in their active groups, even though the molecules themselves are chemically different [16]. Stereospecific similarities in the active groups of the toxins indicates that the problem with cross-reactivity might not be limited to STX and TTX analogues. Later Huot et al. reported the production of monoclonal antibodies that recognized TTX, but not STX [17]. Further investigation and optimization of immunological methods based on anti-TTX antibodies has resulted in the development of more sensitive configurations of standard methods and reduce of non-specific binding of antibodies [18]. Nevertheless, low cross-reactivity of anti-TTX antibodies with TTX-like compounds should be considered when interpreting the results of immunological studies.

Physicochemical methods based on the combination of chromatography and spectrophotometry are frequently used for qualitative and quantitative TTX analyses. High-performance liquid chromatography (HPLC) and thin-layer chromatography (TLC) are commonly used for the substance’s separation. The most common methods are high-performance liquid chromatography with fluorescence detection (HPLC–FLD) and high-performance liquid chromatography followed by mass spectrometry (HPLC–MS). HPLC–FLD allows separating and detecting of both TTX and its analogues. However, substantial differences in the fluorescence intensity of TTX analogues and background signals from the matrix makes this method ineffective for native extracts analysis [19]. HPLC–MS is more sensitive and reliable then HPLC–FLD both in terms of its sensitivity, since the former combines the separation power of the liquid chromatography, and the precision of mass spectrometry to selectively identify and validate the molecular identity of substances [11]. For toxin determination in extract, which is difficult to purify and identify by the other types of analysis, gas chromatography mass spectrometry (GC–MS) is applied [11]. Molecular spectroscopy methods, such as ultraviolet spectroscopy [20], are also used to identify TTX after its extraction. However, due to their low specificity these methods are not common. Electrophoresis is sometimes applied to determine the purity of TTX [21]. Due to the low concentrations of the toxin generally detected in bacteria, physicochemical methods such as TLC, HPLC–FLD, electrophoresis, and spectroscopy lack the required specificity and precision, and therefore cannot be used without follow up analysis. HPLC–MS, HPLC–MS-MS and GC–MS are more sensitive and specific. Due to the complex composition of the matrix and the insolubility of TTX in organic solvents, GC–MS is less suitable for TTX detection. C9 base, which is generally used in this method, is common for several C9 derivatives, making it a less specific indicator of TTX presence.

Another promising method used to evaluate the potential of a given bacteria to produce TTX is to search for the genes involved in TTX biosynthesis using PCR analysis [22]. This method is less labor intensive, allows processing of a large number of samples simultaneously and does not require a large biomass of bacterial cells for analysis. Furthermore, it allows identification 202 of TTX producers among uncultivable microflora. However, lack of data on the TTX biosynthesis pathways and the availability of only putative genes involved in this process at present make this method unattractive.

In a number of studies of bacterial TTX production, the combination of bioassay (using either mice or neuroblastoma cell culture) with chromatographic methods (HPLC–FLD, TLC, GC–MS) was used (Table 2). Liquid chromatography with various mass spectrometry variants (MS, ESI–MS, FAB–MS, MS–MS, MALDI–TOF MS) sometimes was combined with ELISA (Table 2). Electrophoresis, immunohistochemistry, and ultraviolet spectroscopy were less frequently applied for toxin detection in bacterial samples (Table 2).

Application of different methods may give conflicting results. e.g., Matsumura employed HPLC–FLD, GC–MS, monoclonal anti-TTX antibodies, and mouse bioassay for TTX detection in cells of *Vibrio alginolyticus* isolated from *Takifugu niphobles* [23]. The author had shown that the bacterial extracts gave TTX peaks in chromatographic analyzes and positive results in mouse bioassay, but a neutralization test with anti-TTX antibodies was negative. Moreover, polypeptone and yeast extracts, used as a medium for culturing bacteria, gave rise to a chromatographic peak with a retention overlapping that of a TTX control, although TTX-like compounds in the medium materials were not present. Matsumura [24] concluded that application of analytical methods relying on alkali hydrolysis (thin-layer chromatography, electrophoresis, HPLC and GC–MS) for TTX detection in bacteria needs to be verified. Similar results were obtained by another research group [25]. They did not reveal any TTX-producing capacity among *V. alginolyticus* strains isolated from ribbon worm *Lineus longissimus*. However, an active compound (<5 kDa), the paralytic effect on the shore crab of which closely resembled that of TTX, was revealed in the ribbon worm mucus. Thus, using bioassays allows other toxic compounds to be wrongly assumed to be TTX.

To date, LC–MS–MS can be considered the most reliable indicator of TTX molecules’ presence in bacterial samples. This method does not need to be combined by additional analyses. LC–MS is widely used for TTX detection; however, detection of substances with similar molecular weight to TTX in bacterial extracts of *V. alginolyticus* and bacterial media components [23,25] indicates that LC–MS analyses should be supplemented by immunological methods or methods based on the blocking of voltage-gated sodium channels (for example TCBA). As Matsumura [23] and Strand et al. [25] showed, MBA is not a reliable method for the confirmation of TTX presence in bacterial extracts. Such physicochemical methods as LC–FLD, TLC and GC–MS do not allow separation of TTX from its analogues and are less specific to the toxin. To avoid false positives, these methods also should be combined with immunological or voltage-gated sodium channels blocking methods. UV-VIS is not suitable for TTX detection without pure compound as a control, but it can be used for TTX quantification after toxin presence confirmation by another methods. Since there are studies where TTX was detected only by immunological methods [48,57], the question remains whether their usage is sufficient to confirming TTX presence. Nevertheless, for greater reliability of the results, immunological methods should be supplemented by physicochemical methods.

## 3. Ecology of Marine TTX-Producing Bacteria

Discovery of TTX-producing microorganisms is directly linked with the study of TTX-bearing animals, which often become the cause of food poisoning. Noguchi et al. [26] first showed the presence of TTX-producing symbiotic microflora in TTX-bearing animals. The authors isolated bacteria of the genus *Vibrio* from the intestine of the crab *Atergatis floridus* and detected TTX and anhydro-TTX in a cellular extract and culture medium by HPLC–FLD and GC–MS methods. In the same year, TTX-producing strain *Pseudomonas* sp. was isolated from the red alga *Jania* sp. [27]. In recent work, TTX analogues produced by toxic microalga *Prorocentrum minimum* were found to be related to symbiotic bacteria from genera *Roseobacter* and *Vibrio*. [62].

In subsequent years, great interest in the study of bacterial symbionts of marine TTX-bearing animals and their ability to produce the toxin was observed. From 1987 to 1995, TTX-producing bacteria were isolated from a number of animals collected in the Pacific Ocean: from the intestine of the starfish *Astropecten polycanthus* [28]; from several puffer fish species (*Takifugu vermicularis vermicularis* [29], *T. poecilonotus* [31], *T. niphobles* [34]); from the intestine, salivary glands, and tentacles of *Octopus maculosus* [33]; from the intestine of the horseshoe crab *Carcinoscorpius rotundicauda* [32]; from planktonic chaetognaths *Flaccisagitta lyra*, *Parasagitta elegans*, *Zonosagitta nagae*, and *Eukrohnia hamata* [35]; and from muscle and digestive glands of marine gastropods *Natica lineata* [39] and *Niotha clathrata* [40]. Later, TTX-producing bacteria were found in sea urchin *Meoma ventricosa* from the coastal waters of the Caribbean [42].

Most of the studies of the last 15 years have been dedicated to the symbiotic microflora of the puffer fish [49,50,51,52,53,54,55,56,58,59], but TTX producers were also found in several nemertean species [43,57], in sea urchin *Meoma ventricosa* [42], in a copepod *Pseudocaligus fugu* [47], a puffer fish parasite, in a marine gastropod *Nassarius semiplicatus* [48], in mussels *Mytilus edulis* and oysters *Crassostrea gigas* [61], and in a goby fish *Yongeichthys criniger* [60]. Rodríguez et al. [62] identified several *Vibrio* and *Pseudomonas* bacteria, which had previously been reported in TTX production, in contaminated shellfish samples collected in Greece. Most of the TTX-producing strains found in the puffer fish were isolated from the intestine and ovary (63%); others were isolated from the liver (13%), skin and slime on the skin surface (17%), and the total extract of the internal organs (7%).

In 1988, Kogure et al. [12] showed high TTX concentration in marine sediments: 10 g of certain sediment samples contained one mouse lethal dose of the toxin. Both deep-sea sediments (4033 m depth) and sediments from the coastal zone (21 and 81 m depths) contained TTX. The toxin concentration in various samples did not differ significantly [12]. Twenty-eight strains of TTX-producing bacteria of eight genera were isolated from deep-water sediment samples [36,37]. Isolation of typical terrestrial bacteria, such as actinomycetes, bacilli and micrococci, from marine sediments resulted in the investigation of freshwater sediments for the presence of the microbial TTX producers. Seventeen TTX-producing bacterial strains of five genera were isolated from freshwater sediments [38]. The concentration of TTX in the freshwater sediments was one order of magnitude lower than in marine sediments, while the taxonomic diversity of TTX-producing microorganisms was also inferior [38]. TTX production was also revealed for a number of typical marine collection strains of bacteria [30]. Among all of the TTX-producing bacteria described to date, *Pseudoalteromonas tetraodonis* isolated from puffer fish and red algal was not found as a free-living form [63,64]. The occurrence of free-living microorganisms capable of TTX synthesis indicates that the symbiosis is not an essential factor for this toxin production.

Different active biological compounds of bacterial origin, including TTX, are often used by metazoan for various needs [65]. According to the popular assumption, TTX-bearing organisms use toxin for the defense against predators. Okita et al. [66] showed that the survival rate of puffer fish (*T. rubripes*) fry grown under artificial conditions was significantly lower than the survival of fry from the natural environment. The authors explained the low survival rate of the cultured fry by nonadoptive behavior and low TTX concentration (below detectable level) in comparison with the fry from natural habitat. In experiments in which non-toxic cultured puffer fish *T. rubripes* was fed with a TTX-containing diet, Honda et al. [67] revealed that TTX has an immunostimulating effect. Some gastropods secrete TTX in the environment in response to an external stimulus (electrical stimulation) [68,69]. Some species of marine worms, octopuses, and other predatory animals use TTX for hunting [70]. TTX can also serve a communicative function: at least one species of puffer fish (*T. niphobles*) uses TTX as a pheromone [71].

Discovery of the vast majority of TTX-producing microorganisms in the tropical and subtropical regions of the Pacific coast of Asia (mainly in Japan and China) is associated with the active study of TTX-bearing animals in these regions (Table 2). Such geographic distribution of TTX-producing bacteria is largely due to the taxonomic diversity of the animals with which they associate. Due to the high competition among animals living in these regions, some of them focus on a symbiosis with toxin-producing bacteria. However, TTX-producing strains were isolated from several nemertean species [43,57] and bivalve mollusks [61] living in boreal latitudes. In recent years, TTX-bearing puffer fish [72,73], gastropods [74,75,76,77,78], and bivalve mollusks [61,62] were found in the Mediterranean Sea and the Atlantic Ocean. Rising global ocean temperatures and migration of species living in the Red Sea through the Suez Canal are assumed to be the main reasons for TTX-bearing animals’ appearance in European waters [2]. However, it should be noted that temperature increase or decrease may influence growth rate, metabolism, and the diversity of symbiotic microorganisms. Studies of the microbial composition of various organs of the puffer fish *T. niphobles* showed that temperature decrease reduced the diversity and survival of associated bacteria [79]. High TTX concentrations detected in puffer fish *Lagocephalus lunaris* from January to March at a water temperature of 25–26 °C coincided with increased toxin production by symbiotic bacteria *Shewanella putrefaciens* during the same period [56]. In the other months, at a water temperature of 29–30 °C, active growth of this strain was observed, but toxin production by bacteria and toxin concentration in puffer fish were significantly reduced.

## 4. Taxonomic Diversity of the TTX-Producing Bacteria

**Figure 1:**
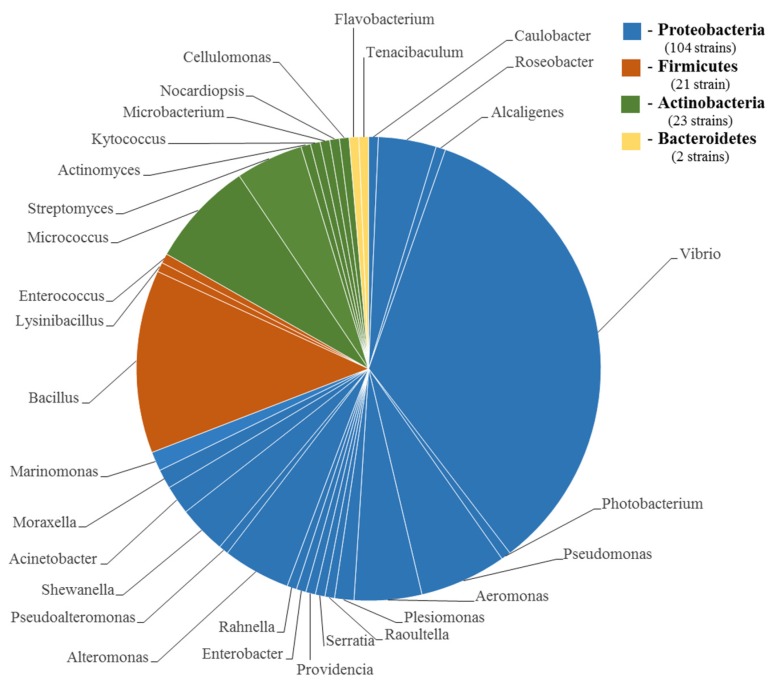
Taxonomic composition of TTX-producing bacteria. The diagram is based on the analysis of 150 TTX-producing strains found to date.

In research on TTX-bearing organisms from both marine and freshwater sediments, TTX-producing representatives of 31 genera of bacteria were isolated (Table 2). Most of the isolated strains belonged to the phylum Proteobacteria and class Gammaproteobacteria (genera *Vibrio*, *Aeromonas*, *Pseudomonas*, *Shewanella*, *Alteromonas*, etc.), but representatives of Alphaproteobacteria (genera *Caulobacter* and *Roseobacter*) and Betaproteobacteria (genus *Alcaligenes*) classes were also found (Figure 1). TTX-producing bacterial strains of the phyla Firmicutes (genera *Bacillus*, *Lysinibacillus* and *Enterococcus*), Bacteroides (genera *Flavobacterium* and *Tenacibaculum*), and Actinobacteria (genera *Actinomycetes*, *Microbacterium*, *Micrococcus* and *Nocardiopsis*) were less frequent.

In the literature, there are reports of at least 150 TTX-producing bacterial strains (Table 2). Representatives of genus *Vibrio* found in many TTX-bearing animals comprise more than 30% of all TTX-producing strains. Most studies indicate a connection between toxin production and the presence of *V. alginolyticus* in the aquatic animals’ microflora [28,29,35]. Representatives of the genus *Bacillus* comprise approximately 15% of the isolated TTX-producing strains. *Pseudomonas*, *Aeromonas*, *Alteromonas*, *Streptomyces*, and *Roseobacter* strains comprise up to 7% of the TTX-producing bacteria. Other genera are only represented by a single strain each.

For the majority of the TTX-producing strains their generic position has been determined; only 25% of isolated strains were identified as particular species (Table 2). Species specificity of bacterial TTX synthesis currently remains an open question. As Matsumura [23] and Strand et al. [25] have demonstrated, investigations on *V. alginolyticus* showed not all strains of this species are able to produce TTX. Similar studies on other species, with some TTX producers among them, have not yet been carried out; therefore, we cannot exclude the possibility that only certain strains have the ability to synthesize the toxin.

## 5. TTX-Producing Bacteria as a Promising Source of the Toxin for the Pharmaceutical Industry

Due to its unique structure, TTX and its analogues block intermembrane currents in excitable tissues (muscular and nervous) without disturbing surrounding tissue homeostasis. This toxin property is widely used in neurobiology [80,81]. The strong analgesic effect of TTX determines its successful application in medical practice. Drugs based on TTX have been tested in clinical trials and can be used as general and local analgesics [82], as well as for local anesthesia [83]. The toxin is of pharmaceutical interest as a neuroprotective agent in ischemic brain injury caused by stroke, and as a renoprotective and antinociceptive agent [59].

Currently, the only method of TTX manufacturing is based on toxin extraction from toxic organs of the puffer fish. This method has low effectiveness and has a negative impact on the aquatic ecosystem, undermining populations of TTX-containing fish of the family Tetraodontidae [84]. Successful attempts at chemical synthesis of this toxin are also known, but these led to low final product yield [85]. Usage of bacterial producers in drug production is more profitable and is the cause of great interest in marine bacteria as a TTX source.

TTX levels detected in bacterial culture of TTX producers seem too low to explain the toxin concentration in TTX-bearing animals [82]. Low substance production can be compensated for by high growth rates and rapid biomass and metabolic products accumulation, typical for bacteria. However, the absence of a model system suitable for TTX synthesis is a major problem for the development of industrial TTX production using bacteria cultivation. Several studies showed that TTX production in bacterial culture was lost after several passages, and bacteria were often transformed into uncultivated forms [48]. To date, only strain *Bacillus* sp. 1839 isolated from nemertean *Cephalothrix simula* [57] has demonstrated toxin production for a long time; this production persisted through multiple cultivation passages. Since researchers [57] have used immunoelectron and immunofluorescent microscopies with anti-TTX antibodies, which can cross-react with TTX analogues [14,15], the question remains which of the TTX-like toxins are produced by the strain. Carroll et al. [43] suggested that the absence of some substances secreted by the host organism might be the reason for the loss of bacterial TTX synthesis. Growth conditions and external factors, such as temperature, medium pH, salinity, etc. are also known to affect bacterial metabolite production. Study of the bacterial strain *Shewanella putrefaciens*, isolated from puffer fish, showed that toxin production depends on the culturing temperature: at 25 °С TTX production by the microorganism was higher than at 30 °С [56]. It was also shown that TTX production depends on the growth phase of the bacterial culture. Yu et al. [55] discovered that toxicity of the 24-h culture of bacteria *Raoultella terrigena* in its active growth phase was almost twice that of the 48-h culture, where cells were in the stationary phase. TTX production by a bacterial strain *Bacillus* sp. 1839—associated with spore formation, while in vegetative cells the toxin was not detected [57,86]. Obtained interesting results on strain *Aeromonas* sp. Ne-1: TTX concentration in a culture medium correlated with the number of copies of the pNe-1 plasmid carried by the bacterial cells [87].

In short, lack of optimal conditions for bacterial cultivation and TTX synthesis, and rapid loss of the ability to produce the toxin, prevent usage of bacteria in industrial toxin production for pharmaceutical manufacture and complicate the search for new TTX-producing strains.

## 6. Discussion

Since discovery of the first bacterial TTX-producers by Noguchi et al. [26], more than 150 TTX-producing strains of various taxonomic groups (Proteobacteria; Firmicutes; Actinobacteria; Bacteroidetes) have been found (Figure 1). The toxin-producing strains were predominantly isolated from the marine environment, including associative/symbiotic microflora of TTX-bearing organisms and marine sediment (Table 2). If the fact of toxin production by marine bacteria is not under discussion, TTX migration in the marine environment raises many questions.

**Figure 2:**
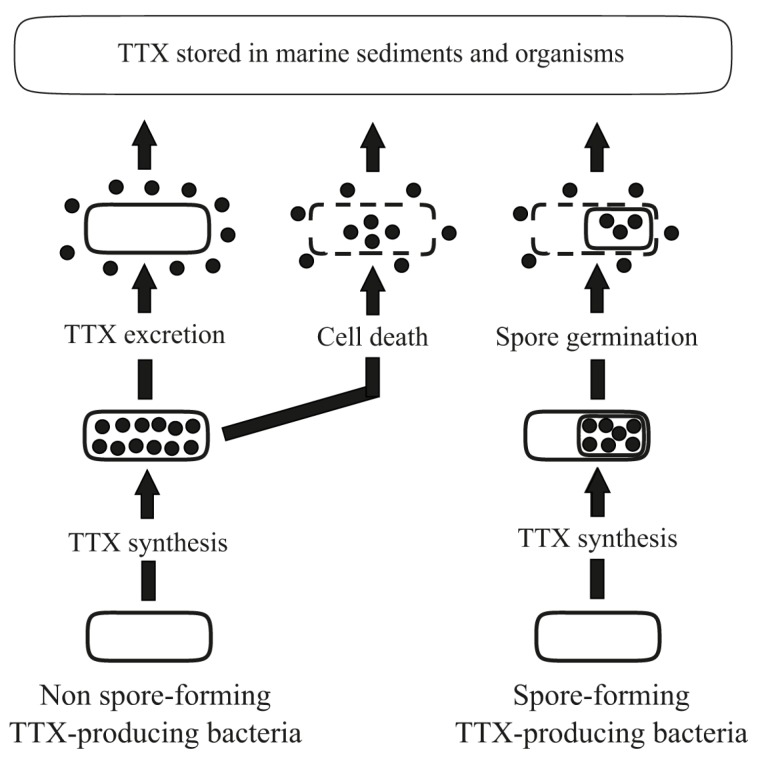
Proposed mechanisms of TTX migration from toxin-producing bacteria to the environment.

The mechanism of TTX extraction from toxin-producing bacteria to the environment is assumed to occur through: (1) cell death; (2) passive/active toxin excretion from cells; (3) spore germination of spore-forming bacteria (Figure 2). Currently the process of bacterial TTX excretion to the environment has been proposed only for representatives of the genus *Bacillus* [57]. Detection of TTX within the cell wall and cores of forespores and free spores of *Bacillus* sp. 1839 led to the suggestion that free and mature spores released from the sporangium of spore-forming bacteria led to toxin release in the environment. Several studies showed TTX appearance both in culture broth and cells during cultivation of *Vibrio* strains in liquid medium [28,35,48]. Revealing the toxin function in bacteria will help to answer the question of whether the migration of TTX in bacterial cells is directional or has a spontaneous nature.

Williams [70] hypothesized that wide distribution of TTX among microorganisms indicates an important role of the toxin for the bacteria, but this role is unknown. The existence of free-living TTX-producing bacteria refutes the main hypothesis, of TTX as a secondary metabolite secreted for the host’s purposes [1]. The search for the molecular targets of a given substance is one of the methods for their functional assignment. The sodium channels of an animal’s excitable tissues is the only known TTX target class [88]. Thus, TTX in a bacterial cell may be a regulator of ion flow driven through voltage-gated sodium channels. To verify this statement, the TTX sensitivity of voltage-dependent sodium channels of toxin-producing bacteria should be investigated. Currently, TTX sensitivity for only two bacterial voltage-dependent sodium channels out of 500 known homologues [89] has been verified. Both channels were isolated from nontoxic alkalophilic bacterium *Bacillus halodurans* and had high resistance to the toxin [90].

TTX-producing bacteria have been predominantly extracted from TTX-bearing animals’ organs in contact with the environment: skin, body wall, gonads, intestines, salivary glands, etc. [33,45,48]. All of the “infected” organs, except intestines, contained high TTX concentration; that is, these organs are “targets” for TTX adsorption. Many authors have proposed that the toxicity of different organs of TTX-bearing animals is associated with the activity of toxin-producing microflora inhabiting these organs [44,51]. Yet data on toxin microdistribution in TTX-bearing animals indicates TTX migration from the digestive system to target organs through the transport system [91,92,93]. In the integument of puffer fish [91,94], Japanese newt *Cynops pyrrhogaster* [95], several nemertean species [96,97], mollusk *Pleurobranchaea maculata* [93], and in the salivary glands of the blue-ringed octopuses (*Hapalochlaena* sp.) [92], the toxin accumulated in the glandular cells, which secrete substances including TTX onto an epithelial surface or into the body cavity. Immunoelectron studies of the ribbon worm *Lineus alborostratus* showed that TTX was localized in the nuclear envelope, endoplasmic reticulum membrane, and secretory granules of TTX-positive gland cells; that is, the toxin was associated with the excretory system of the cells [97]. The epithelial gland cells take up different compounds from transport systems, which include blood and lymphatic systems, the coelomic cavity, parenchymal tissue, etc. The role of transport systems in TTX transfer to target organs has been confirmed for puffer fishes [98,99,100]. For example, TTX administered to nontoxic puffer fish *T. rubripes* intravenously accumulated predominantly in the skin and liver within 12 hours [98]. Only one way of TTX transferring into the transport system through the intestine has been proven to date [98,99,100]. TTX administered into the gastrointestinal tract of nontoxic puffer fish *T. rubripes* was detected in the blood within 30 minutes [98]. Thus, microbial TTX-producers inhabiting the intestine along with TTX-containing food can contribute to organism toxicity via toxin excretion into the intestinal lumen, where toxin enters into the body. The role of TTX-producing bacteria living in the skin, salivary glands, liver, etc. in host toxification remains unknown.

Discovery of the first TTX-producing strain from the intestines of crab *Altergatis floridus* [26] allowed hypothesizing toxification of organisms through microflora inhabiting the alimentary tract. This hypothesis has been confirmed by numerous studies of the intestinal symbiotic microflora of TTX-bearing animals [28,41,55]. Low toxin concentration produced by bacteria in laboratory conditions engendered doubt in host organism toxification through intestinal microflora. Noguchi et al. [101] proposed TTX accumulation through the food chain: TTX both from free-living bacterial producers and dead TTX-bearing organisms is accumulated in sediments; then, through detritus feeders, the toxin migrates through the links of the food chain. Kogure et al. [102] detected high TTX concentration in total tissue homogenate of nematodes inhabiting marine sediment; the toxin concentration was significantly higher than in the puffer fish. Bacteria-feeding organisms such as nematodes have been assumed to be a link between TTX-producing bacteria and other TTX-bearing animals. Another proposed means of TTX introduction into the food chain is through the filter feeders that accumulate toxins produced by unicellular algae. Vlamis et al. [78] detected for the first time TTX in *Mytilus galloprovincialis* from the Mediterranean Sea, and proposed a link between the presence of the toxic dinoflagellate *P. minimum* in seawater and that of TTX in bivalves. Additionally, Rodríguez et al. [62] pointed out that C9-based TTX-like compounds found in *P. minimum* could be precursors of TTX and associated with bacterial production. TTX accumulation at the end of the food chain may happen via highly toxic carnivorous invertebrates’ consumption: such a scenario was described for the super-toxic nemertean *Cephalothrix simula*, which can be eaten by the puffer fish [103]. The contribution of symbiotic TTT-producing microflora to TTX-bearing animals’ toxification, especially at the end of the food chain, in Noguchi’s scheme was insignificant [9].

Many authors point out that a lack of optimal conditions for bacterial growth results in low toxin production by bacterial strains and its further complete loss during cultivation on artificial media [1,43,99,104]. TTX concentration in bacterial culture grown in liquid medium ranged from 42 ng/L for *Vibrio parahaemolyticus* [61] to 0.5–15.7 μg/mL for *Vibrio harveyi* [50]. However, the administration of both low and high TTX concentrations in TTX-bearing animals’ bodies results in an increase of their toxicity. Thus, as Noguchi et al. [101] showed, long-term administration of minimal TTX concentrations (0.5 MU TTX per g of body weight of the animal, which roughly corresponds to 89 ng TTX per g of body weight) through the intestinal tract to non-toxic puffer fish increased animals’ toxicity after 100 days of the experiment. The administration of maximum TTX concentrations (4 MU TTX per g of body weight of the animal, which roughly corresponds to 712 ng TTX per g of body weight) increased toxicity of the fish to 40 days of the experiment. According to this data, even minimal amounts of TTX, produced by intestinal microflora, is able to contribute to an animal’s toxicity. Some researchers, based on diet experiments carried out on a puffer fish, have suggested a minor contribution, or rather a negligible role, of intestinal microflora in the formation of the toxicity of the animal. Changing the diet of puffer fish from the natural habitat to non-typical food led to a loss of animal toxicity, and returning to a natural diet restored toxicity [101]. As shown for many animals, including fish, a diet change to non-typical food causes changes in the qualitative and quantitative composition of intestinal microflora [105,106]. Diet normalization causes recovery of intestinal microflora [107]. Direct evidence demonstrating the role of intestinal microflora in animals’ toxicity formation are not available to date.

## 7. Conclusions and Future Perspective

Up to now, questions of microbial TTX-producers’ biodiversity and their involvement in animal toxification have been better addressed than questions of TTX biosynthesis and the role of the toxin in bacterial cells. Initial attempts to decode the genetic basis of TTX biosynthesis in bacterial producers have been undertaken recently. Liu et al. [87] have shown a positive correlation between copies of pNE-1 plasmid and TTX concentration in the strain *Aeromonas* sp. Ne-1. Another study revealed the presence of non-ribosomal peptide-transferase, which is presumed to take part in TTX guanidine group synthesis, in TTX-producing bacterial strains [22]. Decoding the biosynthetic pathways and functions of TTX in bacterial cells is crucial for investigating further natural sources of TTX. It is obvious that many microorganisms have the ability to produce TTX, but its synthesis depends on unknown conditions; that is, the toxin will not be constantly detected in bacterial culture. Absence of TTX-positive bacteria in some TTX-bearing animals [108] can be explained by the optionality of TTX synthesis, though the possible existence of uncultivated TTX-producing bacterial strains should also be taken into account.

## Figures and Tables

**Table 1:** Common methods for tetrodotoxin (TTX) detection in bacteria.

Group of Methods	Method		Abbreviation	Sensitivity µg/mL	Limitations
**Bioassay**	Mouse Bioassay		MBA	0.2	low accuracy due to individual variability of biological objects; low specificity; little validation data; difficulties in mice purchasing; ethical issues
	Tissue Culture Bioassay		TCBA	0.5	low specificity, allows determining only total Na-channel blocking toxins concentration in the sample
**Immunological methods**	Enzyme-Linked Immunosorbent Assay		ELISA	<0.001	reveals TTX and its analogues
	Immunohistochemistry		IHC		
**Physicochemical methods**	Thin-Layer Chromatography		TLC	2	low specificity; low selectivity; dose not allow quantitative analyzing; results of the analyses depend on external factors
	Liquid Chromatography	with Fluorescence Detection	LC–FLD	0.001–0.002	low sensitivity; difficulties in TTX analogs (which fluorescence intensity is very different from TTX fluorescence) identification
		with Mass Spectrometry	LC–MS		the possibility of false positive results due to impurities in the medium
		with Tandem Mass Spectrometry	LC–MS–MS		-
	Gas Chromatography with Mass Spectrometry		GC–MS	0.001–0.002	TTX is not a volatile substance, its derivation into a volatile form requires a large number of samples and a lot of processing time; not all TTX analogues are thermostable and can be destroyed during chromatography; less specific to TTX due to the C9-base usage
	Ultraviolet–Visible Spectroscopy		UV–Vis spectroscopy	0.1	low sensitivity; spectrum overlay; low specificity
	Electrophoresis		-	2	low sensitivity; low specificity, method detects a range of substances with a close charge

**Table 2:** TTX-producing bacteria isolated from aquatic systems.

TTX-Producing Microorganism	Number of Strains	Source of Isolation *	Place	TTX Detection Method	Toxins Detected	TTX Concentration (µg/mL)	References
*Vibrio* sp.	1	xanthid crab *Atergatis floridus*	Japan	HPLC–FLD GC–MS	TTX anhydro-TTX	NM	[26]
*Pseudomonas* sp.	1	red alga *Jania* sp.	Japan	HPLC–FLD HPLC–FAB–MS MBA	TTX anhydro-TTX	<10	[27]
*Vibrio alginolyticus*	2	starfish *Astropecten polycanthus*	Japan	HPLC–MS UV–Vis GC–MS	TTX anhydro-TTX 4-epi-TTX	0.08	[28]
*Vibrio alginolyticus*	2	puffer fish *Fugu vermicularis vermicularis*	Japan	HPLC–FLD GC–MS	TTX anhydro-TTX	0.0012	[29]
*Vibrio alginolyticus* ATCC 17749 *Vibrio alginolyticus* NCMB 1903	-	American Type Culture Collection (Rockville, MD, USA) National Collection of Marine Bacteria (Aberdeen, Scotland)	-	HPLC–FLD GC–MS	anhydro-TTX	NM	[30]
*Vibrio parahaemolyticus* NCMB 1902 *Vibrio parahaemolyticus* ATCC 17802	-						
*Vibrio anguillarum* NCMB 1291	-						
*Photobacterium phosphoreum* NCMB 844	-						
*Aeromonas salmonicida* ATCC 14174	-						
*Plesiomonas shigelloides* ATCC 14029	-						
*Pseudomonas* sp.	1	puffer fish *Fugu poecilonotus*	Japan	HPLC–FLD GC–MS	TTX anhydro-TTX 4-epi-TTX	NM	[31]
*Vibrio alginolyticus*	1	horseshoe crab *Carcinoscorpius rotundicauda*	Thailand	HPLC–MS UV–Vis GC–MS	TTX anhydro-TTX	NM	[32]
*Alteromonas* sp.	2	octopus *Octopus maculosus*	Philippines	MBA HPLC–FLD GC–MS	TTX anhydro-TTX	NM	[33]
*Bacillus* sp.	2					0.0015	
*Pseudomonas* sp.	1					0.0025	
*Vibrio* sp.	1					NM	
*Shewanella putrefaciens*	1	puffer fish *Takifugu niphobles*	Japan	MBA HPLC–FLD GC–MS	TTX anhydro-TTX	0.012	[34]
*Vibrio alginolyticus*	1	planktonic chaetognaths: *Flussisagitta lyra*, *Parasagitta elegans*, *Zonosagitta nagae*, *Eukrohnia hamata*	Japan	TCBA HPLC–FLD	TTX	0.28–0.79	[35]
*Vibrio* sp.	1	marine sediments	Japan	TCBA HPLC–FLD	TTX anhydro-TTX	NM	[36]
*Bacillus* sp.	5						
*Alteromonas* sp.	5						
*Aeromonas* sp.	1						
*Micrococcus* sp.	4						
*Acinetobacter* sp.	3						
*Moraxella* sp.	2						
*Streptomyces sp.*	7	marine sediments	Japan	TCBA HPLC–FLD GC–MS	TTX	NM	[37]
*Bacillus* sp.	7	freshwater sediment	Japan	TCBA HPLC–FLD GC–MS	TTX anhydro-TTX 4-epi-TTX	NM	[38]
*Micrococcus* sp.	7						
*Alcaligens* sp.	1						
*Caulobacter* sp.	1						
*Flavobacterium* sp.	1						
*Vibrio alginolyticus*	5	lined moon shell *Natica lineata*	Taiwan	HPLC–MS UV–Vis GC–MS	TTX anhydro-TTX 4-epi-TTX	NM	[39]
*Vibrio parahaemolyticus*	1						
*Aeromonas* sp.	2						
*Pseudomonas* sp.	2						
*Vibrio alginolyticus*	3	gastropod *Niotha clathrata*	Taiwan	HPLC–MS UV–Vis GC–MS	TTX anhydro-TTX	NM	[40]
*Vibrio parahaemolyticus*	1						
*Pseudomonas* sp.	4						
*Aeromonas* sp.	1						
*Plesiomonas* sp.	1						
*Vibrio* sp.	1	puffer fish *Fugu vermicularis radiates*	Korea	MBA HPLC–FLD GC–MS TLC Electrophoresis	TTX anhydro-TTX TDA	NM	[41]
*Pseudoalteromonas* sp.	1	sea urchin *Meoma ventricosa*	Caribbean	IHC	TTX	NM	[42]
*Vibrio* sp.	-	ribbon worms: *Cephalothrix rufifrons*, *Lineus longissimus*, *Lineus ruber*, *Lineus viridis*, *Ramphogordius sanguineus*, *Riseriellus occultus*, *Amphiporus lactifloreus*	England	UV–Vis HPLC–FLD	TTX 4-epi-TTX TDA	NM	[43]
*Microbacterium arabinogalactanolyticum*	1	puffer fish *Chelonodon patoca*, *Takifugu alboplumbeus*, *Takifugu niphobles*	Hong Kong	MBA TLC LC–ESI–MS	TTX anhydro-TTX	0.042	[44]
*Serratia marcescens*	1					0.04	
*Vibrio alginolyticus*	1					0.03	
*Bacillus* sp.	3	puffer fish *Fugu rubripes*	China	MBA TCBA TLC HPLC–FLD LC–ESI–MS	TTX	0.32	[45]
*Actinomyces* sp.	1						
*Nocardiopsis dassonvillei*	1	puffer fish *Fugu rubripes*	China	MBA TCBA HPLC–FLD TLC LC–ESI–MS UV–Vis	TTX anhydro-TTX	0.1	[46]
*Roseobacter* sp	6	Copepod *Pseudocaligus fugu*	Japan	MBA GC–MS HPLC–MS	TTX anhydro-TTX	NM	[47]
*Vibrio* sp.	13	gastropod *Nassarius semiplicatus*	China	ELISA	TTX	0.184	[48]
*Shewanella* sp.	3						
*Marinomonas* sp.	1						
*Tenacibaculum* sp.	1						
*Aeromonas* sp.	1						
*Bacillus horikoshii*	1	puffer fish	Taiwan	MBA HPLC–MS HPLC–FLD HPLC–MS–MS	TTX	NM	[49]
*Vibrio harveyi*	1	puffer fish *Arothron hispidus*	Hawaii	HPLC–MS TCBA	TTX anhydro-TTX	0.5–15.7	[50]
*Kytococcus sedentarius*	1	puffer fish *Arothron hispidus*	India	MBA	TTX	NM	[51]
*Cellulomonas fimi*	1						
*Bacillus lentimorbus*	1						
*Bacillus* sp.	1	puffer fish *Fugu obscurus*	China	MBA HPLC–FLD LC–ESI–MS	TTX 4-epi-TTX anhydro-TTX	NM	[52]
*Lysinibacillus fusiformis*	1	puffer fish *Fugu obscurus*	China	MBA LC–ESI–MS	TTX anhydro-TTX	0.024	[53]
*Aeromonas* sp.	1	puffer fish *Takifugu obscurus*	China	MBA ELISA HPLC–MS	TTX	0.002	[54]
*Raoultella terrigena*	1	puffer fish *Takifugu niphobles*	Hong Kong	MBA ELISA LC–MALDI–TOF MS	TTX	0.008	[55]
*Shewanella putrefaciens*	1	puffer fish *Lagocephalus lunaris*	Thailand	HPLC–MS–MS	TTX	0.195–0.366	[56]
*Bacillus* sp.	1	ribbon worm *Cephalothrix simula*	Russia	IHC	TTX	NM	[57]
*Providencia rettgeri*	1	puffer fish *Lagocephalus* sp.	Vietnam	MBA HPLC–FLD TLC	TTX	0.015–0.021	[58]
*Enterococcus faecium*	1	puffer fish	Vietnam	MBA HPLC–FLD TLC	TTX	0.015–0.03	[59]
*Enterobacter cloaca*	1	gobyfish *Yongeichthys criniger*	China	MBA ELISA HPLC–MS–MS	TTX	0.96	[60]
*Rahnella aquatilis*	1					0.6	
*Vibrio cholerae* *Vibrio parahaemolyticus*	1 9	mussel *Mytilus edulis* oyster *Crassostrea gigas*	England	UPLC–ESI–MS–MS	TTX	0.000042–0.000718	[61]

* Species names are listed in accordance with how they are given in the articles. NM = not mentioned; MBA = mouse bioassay; TCBA = tissue culture bioassay; ELISA = enzyme-linked immunosorbent assay; IHC = immunohistochemistry; TLC = thin-layer chromatography; HPLC–FLD = high-performance liquid chromatography with fluorescence detection; HPLC–MS = high-performance liquid chromatography–mass spectrometry; HPLC–MS–MS = high-performance liquid chromatography tandem–mass spectrometry; UPLC–ESI–MS–MS = ultra performance liquid chromatography–electrospray ionisation tandem–mass spectrometry; GC–MS = gas chromatography–mass spectrometry; HPLC–FAB–MS = high-performance liquid chromatography–fast atom bombardment mass spectrometry; LC–ESI–MS = liquid chromatography-electrospray ionisation mass spectrometry; LC–MALDI–TOF MS = liquid chromatography-matrix-assisted laser desorption/ionization time-of-flight mass spectrometry; UV–Vis = ultraviolet–visible spectroscopy.
